# Clinical characteristics of Chinese neonates with neonatal-onset multisystem inflammatory disease: a case report and literature review

**DOI:** 10.3389/fimmu.2023.1291345

**Published:** 2024-01-05

**Authors:** Cui Zhao, Chen Liu, Xiaoying Li

**Affiliations:** Department of Neonatology, Children’s Hospital Affiliated to Shandong University, Jinan, China

**Keywords:** NOMID/CINCA, *NLRP3* gene, novel mutation, neonate, aseptic meningitis

## Abstract

**Background:**

Neonatal-onset multisystem inflammatory disease (NOMID) is a rare and severe autoinflammatory disease caused by mutations of the *NLRP3* gene and is characterized by a skin rash, fever, arthropathy, and neurologic manifestations. We herein report a neonatal case with recurrent rash, fever, and meningitis from 12 h after birth, and NOMID was diagnosed during the neonatal period. We also reviewed the clinical characteristics and genetic mutations of previously reported Chinese neonates with NOMID.

**Case presentation and literature review:**

NOMID is rare in China, and there have been over 100 cases uncovered thus far, including ours. The patient we reported here was the youngest among the confirmed Chinese cases and had the *de novo* mutation c.1210G>C (p.V404L) in exon 4 of the *NLRP3* gene, which has not been reported previously. All 25 patients manifested recurrent urticaria-like rash, and 24 were febrile. Of the 23 patients with genetic data available, all had *NLRP3* mutations. The primary treatment of these patients entailed glucocorticoids and immunosuppressants; however, the IL-1 inhibitor was rarely used due to its current unavailability in China. One patient was cured by umbilical cord blood stem cell transplantation (UCBT), which provided an alternative treatment.

**Conclusion:**

We recommend that NOMID be considered for neonates with recurrent rash, fever, and aseptic meningitis. However, further research on underlying mechanisms and therapeutic regimens in China is necessary to provide improved management.

## 1 Introduction

Neonatal-onset multisystem inflammatory disease (NOMID), also called chronic infantile neurological cutaneous articular (CINCA) syndrome, is a rare autoinflammatory disease that may be grouped into working categories according to its major pathogenesis. Two other conditions referred to as familial cold autoinflammatory syndrome (FCAS) and Muckle-Wells syndrome (MWS)—together with NOMID—are known collectively as cryopyrin-associated periodic syndromes (CAPS), the subserving mechanism of which is associated with disorders of inflammasomes and related interleukin (IL)-1 family cytokines due to mutations in the *NLRP3* gene. The clinical features overlap and include recurrent episodes of unexplained fever, urticaria rash, and joint involvement, with NOMID the severest and rarest form of CAPS. Although characteristics usually appear within the first hours/days of life, they are rarely diagnosed at the neonatal stage, particularly in China, and the youngest patient we found was three months of age. Herein we describe a neonatal case of NOMID with a novel *NLRP3* mutation and present a review of all cases of NOMID reported in China.

## 2 Case presentation

A newborn girl, 15 days of age, was transferred to the neonatal intensive care unit (NICU) of our hospital because of recurrent fever, rash, and abnormal cerebrospinal fluid (CSF) parameters.

The patient was born to a 20-year-old mother by spontaneous vaginal delivery after an uncomplicated gestation of 39 + 3/7 weeks. Membranes ruptured 19h prior to delivery. Because of a rash covering her entire body starting 12 hours after birth, the girl was admitted to the NICU of the local hospital. Initial laboratory results showed a white blood cell (WBC) count of 23.07×10^9^/L (60.9% neutrophils) and a C-reactive protein (CRP) concentration of 23.7 mg/L. Due to the possibility of infection, piperacillin-tazobactam was started empirically, and on day three the baby developed fever; sepsis was then diagnosed, and a lumbar puncture was subsequently performed. CSF showed a WBC count of 292×10^6^/L, a glucose concentration of 2.62 mmol/L (reference range, 2.8 to 4.4), and protein of 1.29 g/L (reference range, 0.15 to 0.45). Since the CSF findings suggested bacterial meningitis, meropenem replaced piperacillin-tazobactam, and intravenous immunoglobulin (IVIG) was administered. However, at day 15, her temperature and rash were not completely controlled, and CRP remained positive, although the CSF WBC count decreased to 112×10^6^/L. The patient was then transferred to our hospital for further evaluation and treatment.

**Figure 1:**
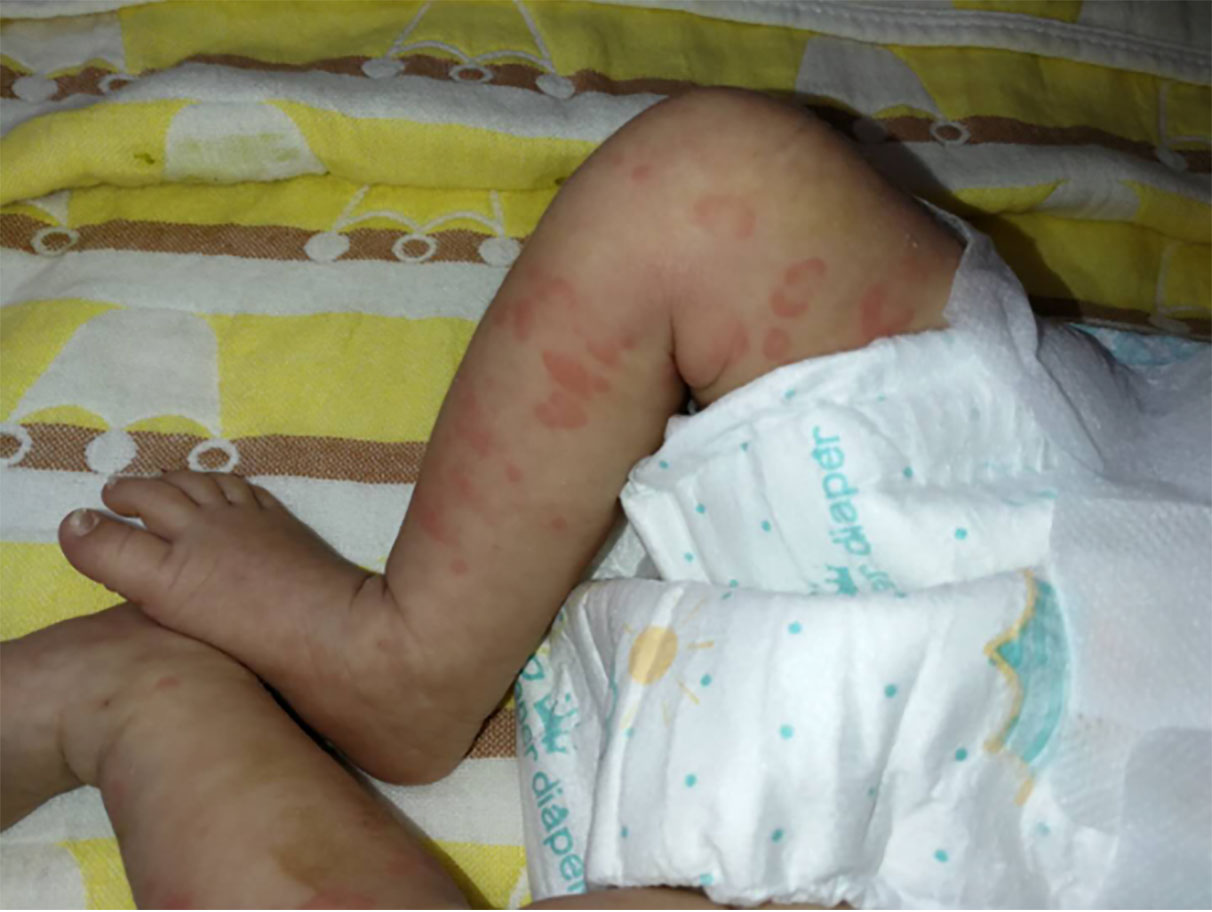
Cutaneous rash in neonatal NOMID patient at 18 days of life.

**Figure 2:**
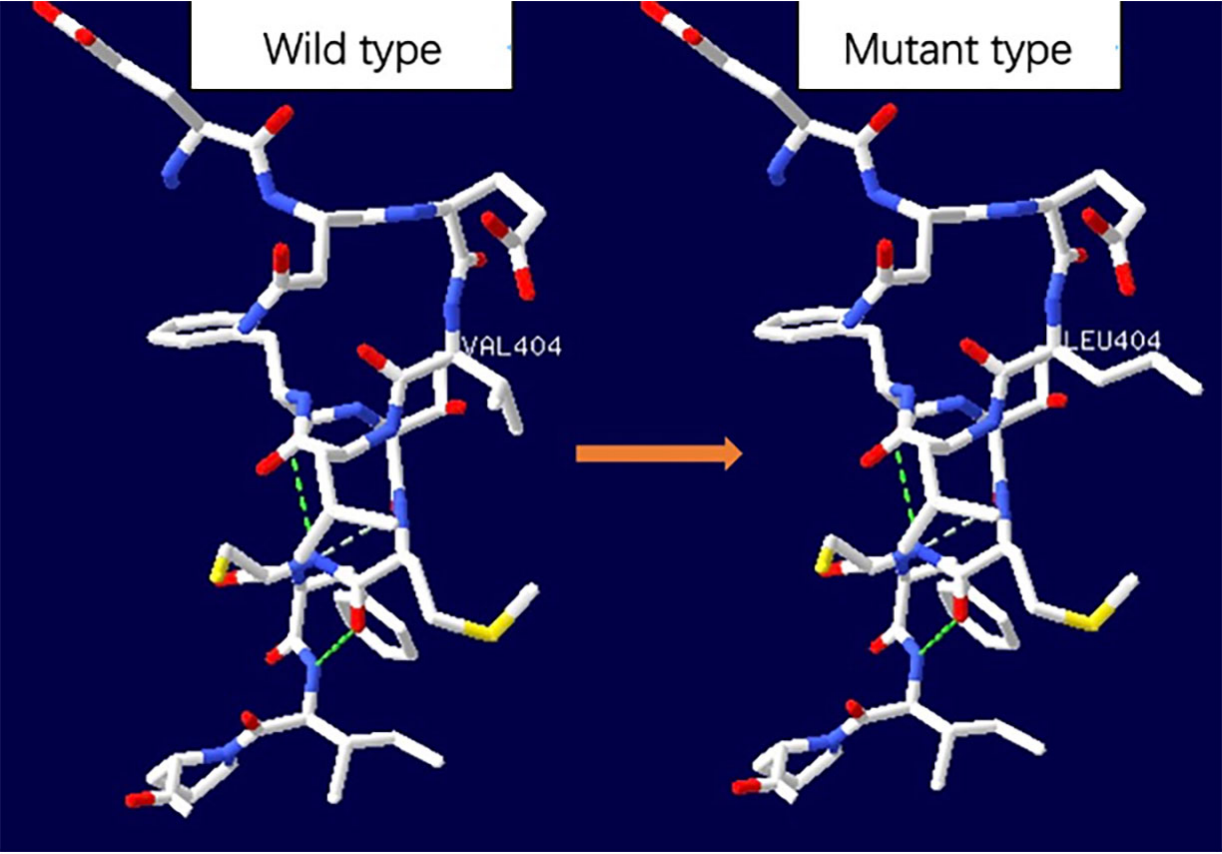
Space distribution of missense variant in NLRP3 protein. The dotted line represented hydrogen bond. No significant hydrogen bond loss was found as predicted by SWISS-MODEL, but the side chain changed.

**Figure 3:**
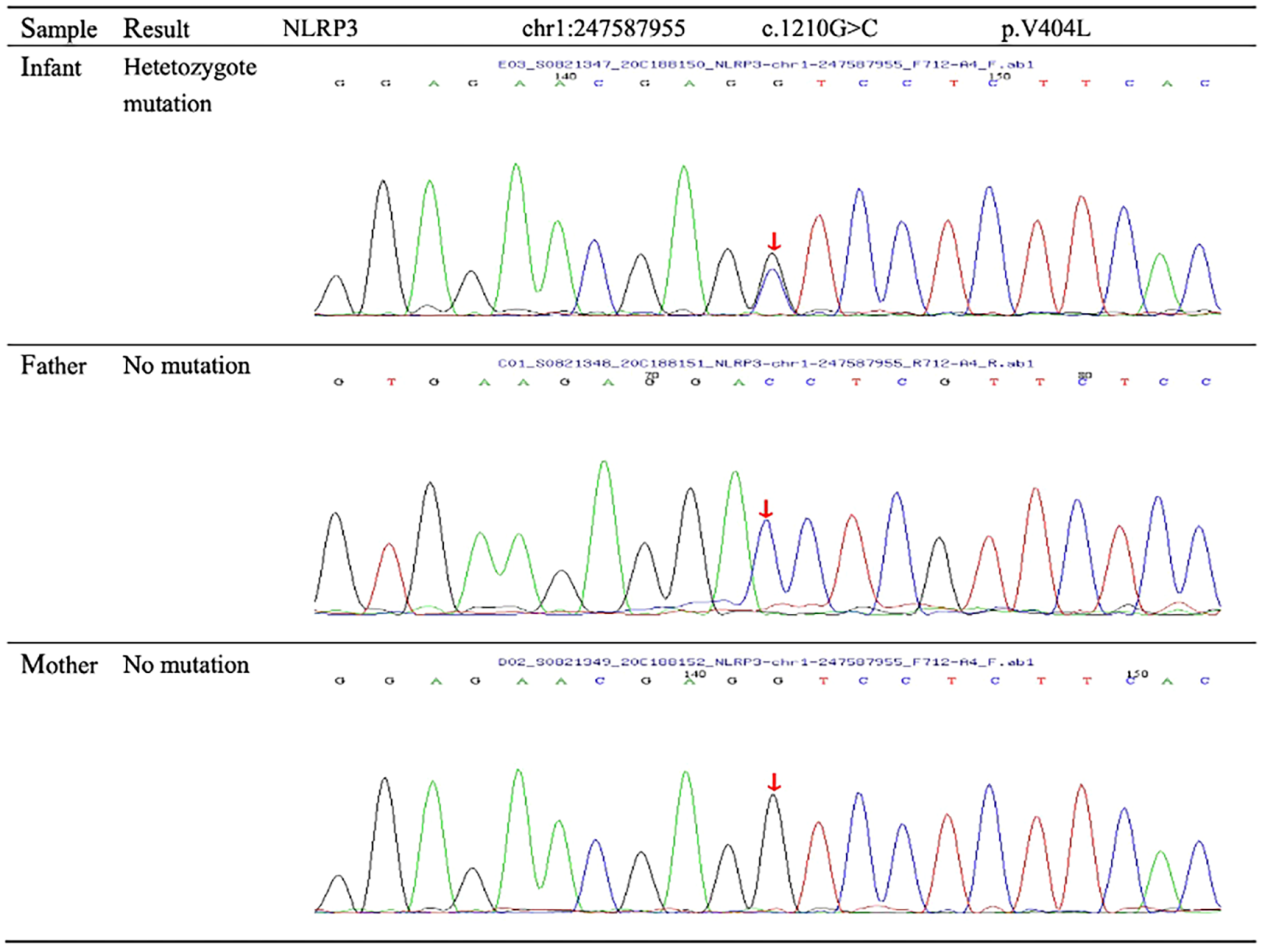
Sequence diagrams for the patient and parents. Genetic testing revealed the patient’s novel missense mutation of c.1210G>C (p.V404L) in the *NLRP3* gene. No mutations of the *NLRP3* gene were found in the parents.

On arrival at the NICU of our hospital, we noted that the patient was afebrile, showed stable cardio-respiratory function, and presented a normal neurologic status. Scattered rashes were still present on her axillary skin. Based on her symptoms and laboratory findings, although the baby was diagnosed with neonatal bacterial meningitis and received meropenem plus vancomycin, treatment was unsuccessful; the neonate still suffered an obvious urticarial-like rash (**Figure 1**) and chronic recurrent fever. In addition, the CSF WBC rose to 1719×10^6^/L, and CRP, erythrocyte sedimentation rate (ESR), and serum amyloid A (SAA) were also elevated. All microbiologic tests were negative, including blood culture, CSF culture, and metagenomic next-generation sequencing (mNGS) of CSF. IgM antibodies to toxoplasmosis, cytomegalovirus (CMV), rubella, and herpes simplex virus (HSV) were negative. Hepatitis B serology was negative. Serum levels of procalcitonin (PCT), (1,3)-beta-D-glucan, alanine aminotransferase, aspartate aminotransferase, direct bilirubin, and coagulation tests were normal—as were the results of antinuclear antibodies. The baby’s electrocardiogram, echocardiography, electroencephalogram, and ophthalmologic evaluations were also normal—as were auditory evoked potentials (AEPs) and visual evoked potentials (VEPs). Cerebral MRI was normal except for enlarged peri-cerebral spaces. Due to the unsatisfactory effect of anti-infection and absence of evidence of infection (including bacteria, virus, fungus, tuberculosis, etc.), autoinflammatory diseases quickly rose to the top of differential diagnosis, particularly when we discovered that dexamethasone could partially relieve the symptoms. Genetic testing ultimately confirmed our suspicion, showing the heterozygote mutation c.1210G>C (p.V404L) in exon 4 of the *NLRP3* gene (**Figure 2**) and the electropherograms of the V404L mutation in samples from the patient, father, and mother are shown in **Figure 3**. The REVEL and PolyPhen-2 software tools were used for prediction of functional effects of this mutation. Although the algorithms showed that the mutation was benign, the variant was *de novo*, it was absent from a large general population by searching publicly available population databases (including the gnomAD v2.1 data set and the 1000 Genomes data set). Therefore, this mutation was determined to be likely pathogenic according to the ACMG Standards and Guidelines. The gene-mutation analysis findings, together with the clinical features, led to the diagnosis of NOMID.

On day 58 (i.e., after 43 days of treatment), the baby was discharged home with a normal temperature, reduced urticarial rash, and normal CRP, SAA, and ESR—although CSF WBC count was still abnormal at 45×10^6^/L. The baby also showed recurrent subtle rashes and mild anemia but a normal temperature and normal acute-phase reactants when we followed up after two weeks of leaving the hospital. Due to the unavailability of anakinra and canakinumab in China and the difficulty to purchase them from abroad due to COVID-19, the baby went to another hospital to receive anti-TNF-a agent (etanercept), which had help partial patients improve symptoms.

## 3 Literature review

We collected previous Chinese reports of NOMID by searching PubMed, Wanfang, and China National Knowledge Infrastructure online literature databases and presented a summary of key baseline indices, clinical manifestations, laboratory features, and genetic analyses (**Table 1**). We have thus far counted 25 NOMID patients from Chinese reports, including our current case and the onset time ranged from 3 hours after birth to 6 years of age, with the age at diagnosis from one month to 16 years. All patients had recurrent urticaria-like rash, and 24 had fever—with other manifestations that included meningitis or CNS manifestations in 22 patients; musculoskeletal involvement in 17 patients; eye involvement in 15 patients; hearing loss in 15 patients; impaired growth in 14 patients; abnormal facies with frontal bossing, protruding eyes, and saddle-shaped nose in 13 patients; cognitive disability in 8 patients; and lymphadenopathy or hepatosplenomegaly in 8 patients. The onset time of the other 24 NOMID patients reported in China ranged from 3 hours after birth to 6 years. Most of them presented clinical features within half one month. Two patients had later onset time: one was 2 years and 5 months old, the other one was 6 years old. They were diagnosed with NOMID not MWS based on the severe clinical features (the frontal bossing, impaired growth, CNS manifestations) and *NLRP3* mutation. Despite the early onset time, the average time at diagnosis was delayed to 6.1 years. Of the 23 cases with genotype available, all exhibited *NLRP3* mutations, including the novel variant c.1210G>C (p.V404L) in our case, which had not been previously reported. One or more drugs were administered to the 25 patients, including corticosteroids, methotrexate, cyclosporine, and thalidomide. Only 2 patients received treatment with canakinumab (one of the anti-IL-treatment medications) to control the symptoms. Two patients were provided etanercept (an anti-TNF-a therapeutic), successfully alleviating clinical symptoms and systemic inflammation. One patient was cured by umbilical cord blood stem cell transplantation (UCBT).

**Table 1:** Clinical symptoms and laboratory findings in 25 Chinese patients with NOMID.

Author, year (Ref)	Number	Age/sex	Age at onset	Fever	Rash	Meningitis/CNS manifestations	Musculoskeletal involvement	Hearing loss	Eye involvement	Mental retardation	Impaired Growth	Abnormal facies	Lymphadenopathy/ Hepatosplenomegaly	Gene analysis
Guan et al. 2014 (1)	1	1year/M	2days	+	+	+	+	+	+	ND	+	+	+	c.1702T>A(F568I)
Fu et al. 2014 (2)	1	20years/F	After birth	+	+	+	ND	+	ND	–	ND	+	+	c.907G>A(D303N)
Zhao et al. 2015 (3)	1	7.7years/M	8months	+	+	–	–	+	+	ND	ND	–	–	c.-2667G>T
Zhang et al.2019 (4)	10	7months-16years (6.4years) /3M	3hours-6years	10/10	10/10	9/10	9/10	8/10	7/10	8/10	10/10	10/10	6/10	c.913G>A(D305N) c.1057G>T(V353L) c.1702T>A(F568I) c.1703T>A(F568Y) c.1710G>C(K570N) c.1789A>G(S597G) c.1991T>C(M664T) c.2269G>A(G757R)
Luo et al. 2020 (5)	2	8months/F 3months/M	2days 1day	+ +	+ +	+ +	ND	ND	+ +	ND	ND	+ ND	ND	c.1568T>A(F523Y) c.1330T>G(F444V)
Jiang et al. 2021 (6)	1	3.5years/M	After birth	+	+	–	+	–	+	ND	ND	ND	ND	c.913G>A(D305N)
Zhou et al. 2022 (7)	8	ND	ND	7/8	8/8	8/8	6/8	4/8	3/8	ND	3/8	ND	ND	c.1715A>G(Y572C)* c.1711G>C(G571R)* c.1991T>C(M664T)* c.1991T>C(M664T)* c.983G>A(G328E)* c.913G>A(D305N)* c.918G>T(E306D)* c.1082T>G(L361W)*
Our case	1	1month/F	12hours	+	+	+	–	–	–	–	–	–	–	c.1210G>C(V404L)*

*The reference sequence was NM_004895; others didn’t provide the transcript number.

## 4 Discussion

NOMID, as the rarest but most severe CAPS phenotype, has been reported in over 100 cases since 1973 when NOMID was first described (4, 8–11), and the first Chinese patient with NOMID was reported in 2014 (1). However, an insufficient understanding and lack of effectively targeted treatment in China often led to permanent organ damage and a poor prognosis (4). We recommend that more doctors (and not only clinical immunologists) pay close attention to this orphan disease because these patients may initially consult various medical providers, including rheumatologists, neonatologists, dermatologists, ophthalmologists, and otolaryngologists.

NOMID patients are generally present at or near the time of birth with recurrent fever, urticarial-like rash, and sustained elevations of acute phase reactants that are easily misdiagnosed as neonatal infections. Intrauterine-onset necrotizing funisitis was recently reported as the first symptom of a newborn with NOMID (12). Although the patients can be identified by typical “facies”, central nervous system (CNS) symptoms, progressive hearing and vision loss, impaired growth, and amyloidosis (8, 13), we expect to be able to make a diagnosis before organ damage occurs. When there is no response to antibiotic therapy, NOMID should be evoked as a differential diagnosis by astute clinicians. In our case, the baby was misdiagnosed with bacterial meningitis initially because of her abnormal CSF, but the diagnosis was questioned since the trend observed for the CSF was irrelevant to anti-infective treatment. Fortunately, we correctly diagnosed the disease with the assistance of clinical immunologists and rheumatologists before the appearance of irreversible organ damage and disability. The onset time of the other 24 NOMID patients reported in China ranged from 3 hours after birth to 6 years; however, the average time at diagnosis was delayed to 6.1 years. Therefore, clinical features such as short stature, intellectual disability, chronic aseptic meningitis, sensorineural hearing loss, eye involvement, lymphadenopathy, hepatosplenomegaly, musculoskeletal symptoms, and skeletal abnormalities are often observed in these patients, but amyloidosis has not been noted thus far. The clinical features noted above have been reported frequently, but there may now be novel unreported complications that relate to the newly described genotype variant of NOMID—including thyroid carcinoma (14). According to a study by Goldbach-Mansky et al. (15), NOMID patients were defined as who present with at least two of the following clinical manifestations: urticarial rash, CNS involvement (e.g., papilledema, pleocytosis in the cerebrospinal fluid, and sensorineural hearing loss), or epiphyseal or patellar overgrowth on radiography. The diagnosis of NOMID can be confirmed by genetic testing for *NLRP3* mutations, but this has not yet replaced clinical evaluation.

*NLRP3* is located on chromosome 1q44 and encodes the NLRP3 protein, also called cryopyrin (16), which serves as a scaffold for the assembly of the NLRP3 inflammasome (17). The NLRP3 inflammasome can make pro-IL-1β and pro-IL-18 into their bioactive forms, then release into the environment (18). Once released, IL-1β elicits neutrophilic inflammation through a cascade of downstream signals. NOMID patients possess the *NLRP3* gene (with point mutations) that encodes aberrant cryopyrin and promotes the formation of the hyperactive inflammasome and inappropriate production of active IL-1β, leading to amplification of inflammation—and even loss of control—following a series of manifestations of organ inflammation. Infevers database (https://infevers.umai-montpellier.fr/) listed 264 *NLRP3* variants in August 2023, and a majority are found in either NOMID or other NLRP3-associated autoinflammatory diseases. The *de novo* missense mutation in our patient was located in exon 4 of the *NLRP3* gene and was not reported previously. *NLRP3* mutations were detected in all the patients with genotype available reported in China. However, approximately 40-65% of NOMID patients lack detectable mutations in the *NLRP3* as determined by Sanger sequencing (8, 19). This is because some of these individuals are carriers of somatic mosaicism, and the proportion is up to 69.2% in patients presenting with symptoms of NOMID and who tested negative by Sanger sequencing (20, 21). Deep sequencing may be needed to detect these somatic mutations to make an accurate genetic diagnosis (22). Low-penetrance *NLRP3* variants may be found in asymptomatic healthy individuals (20, 23), whose management and prognosis may be complicated (22).

Early and effective treatment after making a definite diagnosis is crucial in delaying and preventing organ damage. Since IL-1 plays a central role in NOMID pathogenesis, anti-IL-1 treatment is recommended for NOMID (22). Current anti-IL-1 treatment includes IL-1 receptor antagonists (e.g., anakinra), anti-IL-1β monoclonal antibody (canakinumab), and IL-1 traps (e.g., rilonacept). Although Anakinra has been approved for NOMID by the US Food and Drug Administration (FDA) and for CAPS by the European Medicines Agency (EMA) in 2013 (24, 25), it is not yet available in China. The drug has improved partial signs and symptoms related to inflammation in some (13, 15, 26–28), but not all cases (29), and in severe cases where irreversible lesions developed, its efficacy was greatly diminished (13). The biggest hindrance to long-term compliance with treatment is its short half-life, leading to the regimen of daily injections. Canakinumab, which exhibits a long half-life, improves the acceptability of anti-IL-1 treatment (30). However, it is difficult to achieve full remission for some NOMID patients with severe CNS inflammation, and it is speculated that the penetration of canakinumab into CSF may take longer to reach a steady-state level because of its long half-life (31, 32). The differences in the inhibition of CSF biomarkers between anakinra and canakinumab suggest that anakinra is more effective in the intrathecal compartment (33). Canakinumab has also only been approved by the EMA for patients with NOMID (25), and it is not yet available in China. Another IL-1 inhibitor, rilonacept, is only available in the United States for FCAS and MWS, but there is no evidence supporting its efficacy in NOMID (8, 30, 34). Due to the current unavailability of the aforementioned IL-1-targeted biologics in China, most patients accept conventional anti-inflammatories, immunosuppressants, and antihistamines, but all of these medications have elicited disappointing results (4, 35). High-dose corticosteroids and thalidomide have partially improved symptoms (36), but their administration is always interrupted due to their adverse effects. Some centers have attempted to use anti-TNF-a therapeutic, but only a few animal researches (37) and a few case reports (5) support it. Suffering from occasional fever and subtle rashes, our patient received etanercept in another hospital. The acute-phase reactants were normal before and after the therapy. However, the long-term effects of this treatment on this patient are still needed to be observed. UCBT may be an alternative treatment for children with NOMID in China. Jiang et al. (6) observed that a 4 year old boy with NOMID with a heterozygous mutation of c.913G>A (p. D305N) achieved full remission in fever and urticarial-like rash during a 28-month follow-up after UCBT, and he had no sensorineural hearing loss and eye involvement. Further investigation is required to determine its long-term efficacy. There are now novel treatment approaches under investigation that focus on inhibitors of the NLRP3 inflammasome pathway due to its crucial role in NOMID pathogenesis; these include MCC950, β-hydroxybutyrate, tranilast, autophagy, and microRNAs (38–40).

To increase patient quality of life, Romano M et al. (22) recommend regular monitoring of disease activity to adjust the management strategy, fostering of self-management skills and medical decision-making, and initiating a transition programme to adult specialist care in adolescent patients. Owing to the unavailability of anti-IL-1 therapy in China, the long-term monitoring goals are difficult to achieve. Monitoring for infection is recommended, because patients with NOMID may have an increased susceptibility to pneumococcal pneumoniae. However, pneumococcal vaccines, unlike other vaccines, may lead to exacerbations of inflammation or flare-ups of the disease (41). Hence, clinicians must balance the potential benefits of vaccination against the risks in this population.

In conclusion, the appropriate prognosis of NOMID depends upon early diagnosis and the initiation of aggressive treatment for NOMID patients. Therefore, the features of cutaneous rash, arthritis, and meningitis-like symptoms but ineffectiveness of antibiotic treatment should also be noted by neonatologists—not solely by immunologists and rheumatologists—to initiate treatment of neonates. Specific therapy—especially anti-IL-1 therapies—has proven successful, and recommendations for management have been optimized continuously to improve quality of life. Patients cannot get IL-1blockers from the hospital or chemist’s shop in China. They must purchase them from abroad, and that is difficult for most patients. The high price of IL-1blockers is also another hindrance to long-term compliance with treatment. There are increasing reports of incomplete clinical responses to anakinra and canakinumab emphasizing the need for further understanding of the disease mechanisms and novel drug development. We posit that NOMID patients worldwide should achieve optimal management, including targeted therapy and multidisciplinary cooperation.

## Data availability statement

The original contributions presented in the study are included in the article/supplementary material. Further inquiries can be directed to the corresponding author.

## Ethics statement

The studies involving humans were approved by The Research Ethics Boards of Children’s Hospital Affiliated to Shandong University. The studies were conducted in accordance with the local legislation and institutional requirements. Written informed consent for participation in this study was provided by the participants’ legal guardians/next of kin. Written informed consent was obtained from the individual(s), and minor(s)’ legal guardian/next of kin, for the publication of any potentially identifiable images or data included in this article.

## Author contributions

CZ: Formal analysis, Writing – original draft, Writing – review & editing, Data curation, Methodology, Project administration. CL: Writing – review & editing, Formal analysis, Investigation, Project administration, Resources. XL: Writing – review & editing, Conceptualization, Methodology, Supervision.
